# Changes in the Prevalence of Natural *Paramphistomum* Cercariae Infection in *Indoplanorbis* and *Lymnaea* Intermediate Hosts Influenced by Meteorological Factors

**DOI:** 10.1155/2022/8719834

**Published:** 2022-02-03

**Authors:** Naseem Rafiq, Sultan Ayaz, Sadaf Niaz, Sumbal Haleem, Riaz Ullah, Ahmed Bari, Mohammed Bourhia, Essam A. Ali

**Affiliations:** ^1^Department of Zoology, Abdul Wali Khan University, Mardan, Pakistan; ^2^Department of Zoology, Kohat University of Science and Technology, Kohat, KP, Pakistan; ^3^Department of Pharmacognosy, College of Pharmacy, King Saud University, Riyadh, Saudi Arabia; ^4^Department of Pharmaceutical Chemistry, College of Pharmacy, King Saud University, Riyadh, Saudi Arabia; ^5^Laboratory of Chemistry-Biochemistry, Environment, Nutrition, and Health, Faculty of Medicine and Pharmacy, Hassan II University, Casablanca B.P. 5696, Morocco

## Abstract

Paramphistomosis is a neglected ruminant parasitic disease caused by trematodes known as *Paramphistomum*, which has a diheteroxenic life cycle involving freshwater snail genera, i.e., *Planorbis* and *Lymnaea* as the intermediate host and mammals as the definitive host. Snail vector distribution, infection with *Paramphistomum* spp. cercariae, preferred habitat, and their relationship with certain meteorological factors were not investigated in the province Khyber Pakhtunkhwa of Pakistan. Therefore, this study is designed to evaluate the effects of meteorological factors on the occurrence and severity of *Paramphistomum* spp. cercariae in *Indoplanorbis* and *Lymnaea* intermediate snail hosts. For this purpose, a cross-sectional survey was conducted from October 2018 to September 2019, and snails were collected and then identified using snail shell morphology; their infection with *Paramphistomum* spp. cercariae was determined through microscopy; and descriptive statistics were used to estimate the prevalence of infection and evaluate their occurrence relationship with a certain meteorological factors including temperature, humidity, rainfall, and pan evaporation in different districts of the above-mentioned province of Pakistan, *i.e.*, adjacent areas of Bara and Kabul rivers in district Nowshehra, Kabul River (Sardaryab) of district Charsadda, Kalpani River of district Mardan, and Indus River (Hund) of district Swabi. A total of 2,706 *Indoplanorbis* (1539) and *Lymnaea* (1167) snails were collected, in which overall 10.30% shed *Paramphistomum* spp. cercariae. The highest infection rate was found in the river of district Swabi (13.20%), while the lowest in adjacent rivers of district Nowshehra (8.19%). Meteorological factors play an essential role in the causation of *Paramphistomum* spp. infection, parasitic reproduction, vector growth, and survival. Due to these factors, high significant prevalence was found in the summer season (11.83), followed by autumn (11.25), which might be due to optimum temperature, relative humidity, and rainfall (*p* < 0.05). It was concluded from the study that meteorological factors contribute to the prevalence of *Paramphistomum* species in the *Indoplanorbis* and *Lymnaea,* which act as vectors for the disease paramphistomosis, which may lead to the increased intensity of infection outbreaks of the parasite population in humans and domestic animals.

## 1. Introduction

Paramphistomosis is a neglected ruminant parasitic disease with a broad geographic distribution caused by Trematoda that belongs to the Paramphistomatidae family. The genus name *Paramphistomum* is a derivative of a Greek word amphistomes, meaning paired mouth [1]. The life cycle of *Paramphistomum* involves a diheteroxenic cycle including snails as the intermediate host and mammals as the definitive host. The snail species may be *Lymnaea bulimoides*, *Bulinus* spp., and *Planorbis planorbis* [2]. The shape of a live, mature worm is conical, and the color is pink. Its length ranges from 5 to 12 mm. The front end bears an oral sucker, while there is a large ventral sucker at the rear end. It has a convex dorsal surface and a concave ventral surface [3]. After hatching in water, the egg (miracidia) infects freshwater snails (bulinid or planorbid), which are their intermediate [4]. Adult flukes are usually considered nonpathogenic to their hosts; however, the invasion of immature worms through duodenal mucosa induces serious enteritis, likely necrosis and hemorrhage, and is responsible for anorexia, polydipsia, unthriftiness, severe diarrhea, and mortality in the definitive host. [5]. Paramphistomosis leads to lower conversion of nutrition and a decrease in the production of weight and milk in ruminant grazing [6]. Paramphistomosis prevalence is high in all subtropical and tropical regions, particularly in Russia, Australia, Eastern Europe, Asia, and Africa [5, 7–9]. The prevalence is 30–60% in some regions of Asia including Pakistan [5, 10, 11]. Gastrointestinal helminths, including *Paramphistomum* species, were recorded to be 25.1 to 92 percent in various areas during different times in Pakistan [10, 12–15].

The species of *Paramphistomum* have a complex life cycle that requires freshwater snails as an intermediate host for completion of its life cycle. Therefore, the transmission of paramphistomosis can be minimized by controlling the infection in the intermediate host, as the snails are mobile and able to spread the infection in an ungrazed posture. The prevalence of the *Paramphistomum* species may be less than 2 percent and may grow to 20 percent or sometimes higher if the conditions for hatching of the eggs in the intermediate snail host are sufficient [15].

Prevalence of *Paramphistomum* and its extent depend on specific factors. Various studies show that climate change can modify the geographic distribution of parasite infections and potentially cause drastic changes to their hosts. The life cycle and prevalence of the *Paramphistomum* spp. depend on several meteorological factors. This led to the creation of the prediction method based on meteorological data prevising the timing and severity of the infection [16]. Such predictions will serve as the basis for the system of animal control. Temperature, humidity, rainfall, water velocity, and habitat stability are some of the main factors determining the production of many parasite infections [17]. These factors affect the metabolic system of both the parasite and the host (snail), interfering with the rate of growth, survival, and reproduction of snails [18, 19]. Snails belonging to the genus *Indoplanorbis* and *Lymnaea* are widespread in different locations in Pakistan and act as an intermediate host for the transmission of various parasite infections including paramphistomosis. However, to the authors' best knowledge, no previous study on the prevalence of *Paramphistomum* spp. cercariae in the intermediate snail host was conducted in Khyber Pakhtunkhwa, Pakistan.

In the backdrop of the above discussion, this study is designed to evaluate the effects of meteorological factors on the occurrence and severity of *Paramphistomum* spp. cercariae in *Indoplanorbis* and *Lymnaea* intermediate snail hosts.

## 2. Materials and Methods

### 2.1. Study Design

Duration of the present study was from October 2018 to September 2019, and freshwater *Indoplanorbis* and *Lymnaea* intermediate snail hosts infected with *Paramphistomum* spp. cercariae were collected from different districts including adjacent areas of Bara and Kabul rivers in district Nowshehra, Kabul River (Sardaryab) of district Charsadda, Kalpani River of district Mardan, and Indus River (Hund) of district Swabi of province Khyber Pakhtunkhwa, Pakistan.

### 2.2. *Indoplanorbis* and *Lymnaea* Collection and Identification

For the collection of the *Indoplanorbis* and *Lymnaea* intermediate snail hosts, a scoop net was used, which consists of a metal ring with a shallow dip net attached to a handle used in fishing. The diameter of the ring was 30 cm, while the length was 36 cm. A wire net of 16 m ash per inch was attached to the metal ring. A total of 2,706 snails were collected both from the immersed zones and shallow or stagnant waters of each area. These snails were transferred to polythene bags filled with some water and vegetation and were transported to the Laboratory of Parasitology, Department of Zoology, Abdul Wali Khan University, Mardan, on the same day. The collected snails were kept in water tanks provided with vegetation such as *Vallisneria spiralis* and *Hydrilla verticillata* and constantly aerated. Certain ecological parameters were recorded during snail collection, particularly temperature and pH of the water. Majority of the snails were attached to the back side of the leaves found on the side walls of the pond at the depth of 1–3 feet. The snails were found in shaded areas of ponds. The collected snails were identified to the genus level through keys provided by the guide entitled as “Field guide to the non-marine molluscs of Southeastern Australia” [20].

### 2.3. Examination of *Indoplanorbis* and *Lymnaea* Intermediate Snail Hosts for *Paramphistomum* spp. Cercariae Infection

For the examination of the *Indoplanorbis* and *Lymnaea* intermediate snail hosts for *Paramphistomum* spp. cercariae infection, the snails were placed in water-filled specimen tubes for a period of 24 hours. After regular intervals of 60 minutes, the tubes were observed in light for the presence of cercariae. The snails were regarded infected if encysted cercariae emerged on the walls of the tube. Infected snails were isolated for further examination. Sometimes, for a long time, the snails did not protrude their bodies out of the shell, so the cercariae were not discharged. Such snails were reexamined until the snail shelled out their bodies. The snails were further examined under a stereomicroscope for confirmation of the infection with *Paramphistomum* spp. cercariae. The highly contractile cercariae with a tail were collected for further examination.

### 2.4. Examination of the *Paramphistomum* spp. Cercariae

The collected cercariae were examined under a microscope for the confirmation of *Paramphistomum* spp. infection. The *Paramphistomum* cercariae were identified according to the criteria outlined by Dinnik [21] and Eduardo [22]. The cercariae having *Paramphistomum* spp. rediae are shorter with a small pharynx lacking a collar and appendages in their bodies [23]. These cercariae are dark brown in color and sluggish. The prevalence of natural infection of each *Indoplanorbis* and *Lymnaea* intermediate snail host was determined by dividing the number of snails infected by *Paramphistomum* spp. cercariae by the total number of snails examined.

### 2.5. Meteorological Data Collection

The meteorological factors including pan evaporation, humidity, rainfall, and temperature were obtained from the Regional Meteorological Center, Peshawar, Government of Pakistan, in soft format (the only nearest station in Khyber Pakhtunkhwa, Pakistan).

### 2.6. Statistical Analysis

The data obtained were statistically analyzed in Excel sheets by applying the chi-square test (*χ*^2^) by using SPSS. *P* < 0.05 is considered statistically significant.

## 3. Results

### 3.1. Total Natural *Paramphistomum* spp. Infection in *Indoplanorbis* and *Lymnaea* Intermediate Snail Hosts

Table 1 shows the meteorological data of four adjacent districts of the province Khyber Pakhtunkhwa, Pakistan, during the period of October 2018 to September 2019.

**Figure 1:**
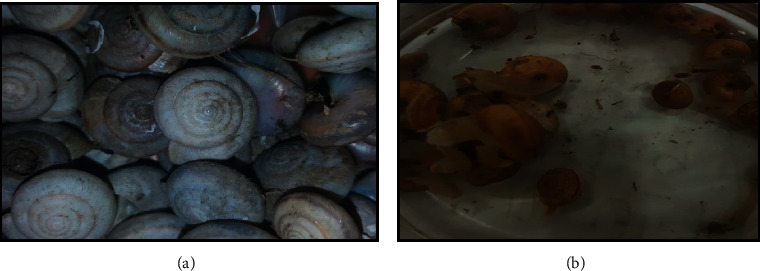
Snails belonging to the genera *Indoplanorbis* collected during the study for the investigation of the prevalence of *Paramphistomum* spp. cercariae (collection site: adjacent area of the river in district Swabi).

**Figure 2:**
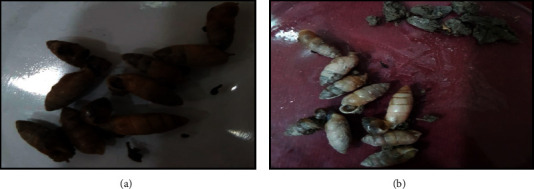
(a, b) Snails belonging to the genera *Lymnaea* collected during the study for the investigation of the prevalence of *Paramphistomum* spp. cercariae (collection site: adjacent area of the river in district Charsadda).

**Figure 3:**
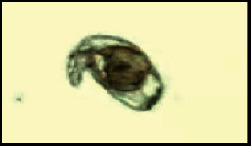
Cercariae (100×) of *Paramphistomum* spp. found in the snail belonging to the genus *Lymnaea*.

A total of 2,706 snails were collected from the rivers of four districts of Khyber Pakhtunkhwa, Pakistan, including districts Mardan, Swabi, Charsadda, and Nowshehra. Of the total snails collected, 1539 belonged to the genus *Indoplanorbis* and 1167 to the genus *Lymnaea* (Figures 1(a), 1(b) and 2(a), 2(b), respectively). The water from the areas of collection had pH between 7.2 and 7.9, and vegetation of *Nuphar lutea* (lily) and *Nelumbo lutea* (lotus) was found abundant in the habitat. In the total collected snails, 10.38% snails of both genera *Indoplanorbis* and *Lymnaea* were found infected with *Paramphistomum* spp. cercariae (Figure 3) with a significant difference *P* < 0.05 (*P*=0.0028, *χ*^2^ = 14.08) (Table 2).

### 3.2. Monthwise and Areawise Natural *Paramphistomum* spp. Cercariae Infection in *Indoplanorbis* and *Lymnaea* Intermediate Snail Hosts

The peak *Paramphistomum* cercariae infection was recorded during July (12.7%), followed by August (12.0%), while no infection was recorded in December (0.00%) and January (0.00%) in the total 839 snails (371 of genus *Indoplanorbis* and 468 of genus *Lymnaea*) collected from the Kalpani River of district Mardan.

Of the total 535 snails collected from the Kabul River (Sardaryab) of district Charsadda, 243 belonged to the genus *Indoplanorbis* and 292 were from the genus *Lymnaea*. The infection rate was highest in October (19.4%), and no infection was recorded in the months of January (0.00%) and February (0.00%).

The prevalence was highest in November (12.5%), and the prevalence of *Paramphistomum* spp. cercariae was not recorded in the months of January (0.00%) and February (0.00%) (Table 2), in the total 696 collected snail samples (303 of genus *Indoplanorbis* and 393 of genus *Lymnaea*) from the adjacent areas of Bara and Kabul rivers in district Nowshehra.

In the Indus River (Hund) of district Swabi, a total of 636 snails were collected, of which 249 belonged to the genus *Indoplanorbis* and 387 to the genus *Lymnaea*, with the highest infection rate in the month of July (26.2%) and lowest in December (3.8%), while no infection was recorded in the months of January (0.00%) and February (0.00%).

Furthermore, during the time of sample collection, the highest infection rate was recorded in July (15.6%), followed by June (13.3%), while no infection was recorded in the months of January (0.00%) and February (0.00%), showing a significant difference in the infection rate in different months (*χ*^2^ = 8.467, *P*=0.0373).

Moreover, the highest total infection rate was recorded in the Indus River (Hund) of district Swabi (13.20%), followed by the Kabul River (Sardaryab) of district Charsadda (11.40%), Kalpani River of district Mardan (9.14%), and adjacent areas of Bara and Kabul rivers in district Nowshehra (8.19%) (Table 2).

### 3.3. Seasonwise Natural *Paramphistomum* spp. Cercariae Infection in *Indoplanorbis* and *Lymnaea* Intermediate Snail Hosts

For the determination of seasonwise natural prevalence of *Paramphistomum* spp. cercariae infection in *Indoplanorbis* and *Lymnaea* intermediate snail hosts, one year was divided into four groups as per season in the respective months. Winter consisted of five months (November 2018–February 2019), the spring season two months (March 2019–April 2019), the summer season four months (May 2019–August 2019), and the autumn season two months (October 2018 and September 2019). The results showed that the infection rate was at the peak during mid-summer (11.83%), followed by autumn (11.25%) and spring (7.07%), whereas the lowest prevalence rate was recorded in the winter season (5.1%), showing a significant difference in the infection in different seasons (*P* < 0.01) (Table 3).

### 3.4. Natural *Paramphistomum* spp. Cercariae Infection in *Indoplanorbis* and *Lymnaea* Intermediate Snail Hosts Influenced by Meteorological Factors

It was found during the study that the prevalence of infection was positively correlated to minimum and maximum temperature, pan evaporation, humidity, and rainfall with highly significant differences. The data showed (Tables 1 & 2) that the disease incidence was highest in June and July when the highest average temperature(32.5°C and 30.7°C) was recorded, the lowest relative humidity was 53.5% and 40.0%, rainfall was 128.0 mm and 122.0 mm, and pan evaporation was 224.3 mm and 200.3 mm, respectively. Moreover, the infection was lowest in the months of January, December, and February when the lowest temperature (11.9, 13.45, and 14.8°C) was recorded, respectively.

## 4. Discussion

Snails play an essential role in the spread and transmission of paramphistomosis by acting as a vector including genera *Indoplanorbis* and *Lymnaea.* In Pakistan, the ecological conditions are appropriate for the survival of various species of snails. During the present study, 2,706 snails were collected from different regions of the province Khyber Pakhtunkhwa and 10.30% were found infected with *Paramphistomum* spp. cercariae.

In all four selected study sites, the highest infection rate was recorded in the Indus River (Hund) of district Swabi (13.20%), which might be due to the reason that crop grown in abundance in the respective area leads to water logging that helps in snail reproduction and breeding and for the metacercariae and cercariae of *Paramphistomum* species. Different environmental conditions and management correlated with the difference of snail prevalence in different locations of the country. Our findings can be co-related with the findings of another study conducted in the district Gujranwala of province Punjab, Pakistan, on the incidence of paramphistomosis in cattle [24, 25]. In a previous study, the environmental data from the farms of origin of the necropsied cows were used in Bayesian geostatistical models to forecast the prospect of infection by *C. daubneyi* throughout the region. These findings confirmed the role of environmental risk aspects in elucidating the geographical heterogeneity in the probability of infection in beef and dairy cattle [26]. A positive correlation of infection rate with pan evaporation, humidity, rainfall, and temperature was observed. The current study findings showed that trematodal cercariae are not found in all seasons but are found in specific times of the year. Two periods of the highest incidence of metacercariae and cercariae of *Paramphistomum* species were observed in the months of July–August and October, whereas the lowest infection rate was observed during the months of December–February. Moreover, the results showed that the infection rate was at a peak during the summer season, which might be due to the factors of optimum temperature, relative rainfall, and humidity. These factors play a significant role in the propagation of the *Paramphistomum* species, and thus a considerable difference was found in the rate of incidence of paramphistomosis in correlation with temperature, humidity, and rainfall in each season, showing agreement with the earlier studies in different regions [24, 25, 27–30] and disagreement to the other studies [31, 32].

## 5. Conclusion

It is the first report on the prevalence of *Paramphistomum* species cercariae in the *Indoplanorbis* and *Lymnaea* intermediate snail hosts in the study area. It was concluded from the study that meteorological factors contribute to the prevalence of *Paramphistomum* species cercariae in *Indoplanorbis* and *Lymnaea,* acting as vectors for the disease paramphistomosis that may lead to the increased intensity of infection outbreaks of the parasite population in humans and domestic animals.

## Figures and Tables

**Table 1:** Mean monthwise temperature (°C), relative humidity (%), rainfall (mm), and pan evaporation (mm) during the study period in the adjacent districts of Khyber Pakhtunkhwa (October 2018 to September 2019).

Time (months)	Temperature (C°)			Relative humidity (%)			Rainfall (%)	Pan evaporation (mm)
	Minimum	Maximum	Average	Morning	Evening	Average		
Oct 2018	17.3	31.9	24.6	82	48	65.0	0.0	78.0
Nov 2018	11.0	22.7	16.85	92	66	79.0	61.0	58.5
Dec 2018	5.9	23.0	14.45	82	52	67.0	12.3	47.1
Jan 2019	3.8	20.1	11.95	83	50	66.5	0.0	44.4
Feb 2019	7.9	23.6	15.75	83	48	65.5	36.5	51.8
Mar 2019	13.8	29.0	21.40	77	46	61.5	23.8	89.8
Apr 2019	18.1	32.5	25.30	76	49	62.5	102.3	102.9
May 2019	22.1	36.1	29.10	63	44	53.5	54.0	182.1
Jun 2019	25.8	41.2	33.50	66	41	53.5	19.0	225.3
Jul 2019	25.6	37.7	31.65	78	62	70.0	121.0	201.3
Aug 2019	26.6	37.1	31.85	80	63	71.5	25.6	167.9
Sep 2019	23.2	36.8	30.00	77	51	64.0	19.0	131.7

**Table 2:** Areawise and monthwise prevalence of *Paramphistomum* spp. cercariae infection in *Indoplanorbis* and *Lymnaea* intermediate snail hosts in various districts of Khyber Pakhtunkhwa, Pakistan.

Areas	Month-year	Snail hosts		Total observed	Infected with *Paramphistomum* spp.	Rate of infection (%)	*χ* ^2^	*P*
		*Lymnaea* spp.	*Indoplanorbis* spp.					
River Kalpani of district Mardan	Oct-18	38	27	65	04	6.1		
	Nov-18	30	13	43	03	6.9		
	Dec-18	10	05	15	00	0.0		
	Jan-19	12	10	22	00	0.0		
	Feb-19	15	11	26	01	3.8		
	Mar-19	29	26	55	06	10.9		
	Apr-19	48	52	100	08	8.0		
	May-19	42	44	86	08	9.3		
	Jun-19	68	42	110	12	110.9		
	Jul-19	54	32	86	11	12.7		
	Aug-19	58	50	108	13	12.0		
	Sep-19	63	60	123	13	10.5		
	Total	467	372	839	79	9.41		

Kabul River (Sardaryab) of district Charsadda	Oct-18	19	17	36	7	19.4		
	Nov-18	22	12	34	3	8.8		
	Dec-18	06	05	11	1	9.0		
	Jan-19	04	03	7	00	0.0		
	Feb-19	04	03	7	00	0.0		
	Mar-19	03	02	5	00	0.0		
	Apr-19	28	19	47	02	4.25		
	May-19	14	11	25	02	8.00		
	Jun-19	23	20	43	04	9.3		
	Jul-19	65	52	117	16	13.6		
	Aug-19	62	58	120	14	11.6		
	Sep-19	42	41	83	12	14.4	8.467	0.037
	Total	292	243	535	61	11.40		

Adjacent areas of Bara and Kabul rivers in district Nowshehra	Oct-18	21	19	40	2	5.0		
	Nov-18	13	11	24	3	12.5		
	Dec-18	10	02	12	1	8.3		
	Jan-19	08	01	09	00	0.0		
	Feb-19	06	1	7	00	0.0		
	Mar-19	20	1	21	00	0.0		
	Apr-19	31	22	53	00	3.7		
	May-19	35	29	64	03	4.6		
	Jun-19	38	30	68	05	7.3		
	Jul-19	85	81	166	17	10.24		
	Aug-19	73	62	135	13	9.6		
	Sep-19	53	44	97	11	11.3		
	Total	393	303	696	57	8.19		

Indus River (Hund) of district	Oct-18	46	39	85	08	9.4		
	Nov-18	32	12	44	06	13.6		
	Dec-18	20	06	26	01	3.8		
	Jan-19	15	05	20	00	0.00		
	Feb-19	19	06	25	00	0.00		
	Mar-19	23	04	27	02	7.4		
	Apr-19	10	07	17	03	17.6		

**Table 3:** Seasonwise prevalence of *Paramphistomum* spp. cercariae infection in *Indoplanorbis* and *Lymnaea* intermediate snail hosts in various districts of Khyber Pakhtunkhwa, Pakistan.

Seasons	Snail hosts		Total observed	Infected with *Paramphistomum* spp.	Rate of infection (%)
	*Lymnaea* spp.	*Indoplanorbis* spp.			
Winter: Nov 2018–Feb 2019	226	106	332	17	5.1%^*∗*^
Spring: Mar 2019–Apr 2019	192	133	325	23	7.07%^*∗*^
Summer: May 2019–Aug 2019	796	640	1463	170	11.83%^∗∗^
Autumn: Oct 2018 & Sep 2019	326	287	613	69	11.25%^∗∗^

^*∗*^Significant (*P* < 0.05), and ^∗∗^highly significant (*P* < 0.001).

## Data Availability

The data used to support the findings are included within the article.
